# Evaluation of retinol binding protein 4 and carbamoylated haemoglobin as potential renal toxicity biomarkers in adult mice treated with ^177^Lu-octreotate

**DOI:** 10.1186/s13550-014-0059-x

**Published:** 2014-10-31

**Authors:** Johanna Dalmo, Emelie Westberg, Lars Barregard, Lisa Svedbom, Martin Johansson, Margareta Törnqvist, Eva Forssell-Aronsson

**Affiliations:** Department of Radiation Physics, Institute of Clinical Sciences, Sahlgrenska Cancer Centre, Sahlgrenska Academy, University of Gothenburg, Sahlgrenska University Hospital, Gothenburg, SE-413 45 Sweden; Department of Medical Physics and Biomedical Engineering, University of Gothenburg, Sahlgrenska University Hospital, Gothenburg, SE-413 45 Sweden; Department of Materials and Environmental Chemistry, Stockholm University, Stockholm, SE-106 91 Sweden; Department of Occupational and Environmental Medicine, Institute of Medicine, Sahlgrenska Academy, University of Gothenburg, Gothenburg, S-405 30 Sweden; Department of Laboratory Medicine, Faculty of Medicine, Lund University, Lund, SE-221 00 Sweden; Clinical Pathology, Skåne University Hospital, Malmö, SE-205 02 Sweden

**Keywords:** Peptide receptor radionuclide therapy, Nephrotoxicity, RBP, Creatinine, Urea, Carbamoylated haemoglobin, Valine hydantoin

## Abstract

**Background:**

The kidneys are regarded as one of the main dose-limiting organs in the treatment of neuroendocrine tumours with ^177^Lu-[DOTA^0^, Tyr^3^]-octreotate (^177^Lu-octreotate), despite the successful use of kidney uptake blocking agents such as lysine and arginine. To avoid renal toxicity but still give each patient as high amount of ^177^Lu-octreotate as possible, there is a need for methods/biomarkers that indicate renal injury in an early stage of the treatment. The aim of this study was to investigate the potential of using urinary retinol binding protein 4 (RBP4) and carbamoylated haemoglobin (Hb) in blood as biomarkers of nephrotoxic effects on adult mice after ^177^Lu-octreotate treatment.

**Methods:**

Adult BALB/c nude mice were injected with 60 MBq or 120 MBq of ^177^Lu-octreotate or with saline (control). Urine was collected before injection and concentrations of urinary RBP4 and creatinine were determined 14 to 90 days after injection Blood samples were collected after 90 days, and carbamoylated N-terminal valine in Hb, formed from urea, was measured as valine hydantoin (VH) after detachment from Hb.

**Results:**

The RBP4 values increased with administered activity and time. For the 60 and 120 MBq groups, statistically significantly higher RBP4 levels (*p* <0.05) were found at day 60 and 90 compared to baseline, also at day 30 for 120 MBq group. For VH, the mean values were similar for the 60 MBq and control groups, while a small increase was observed for the 120 MBq group; but there were no statistically significant differences between any of the groups (*p* >0.05). No morphological changes in the kidney tissue were found.

**Conclusions:**

Urinary RBP4 is a promising new biomarker for radiation-induced renal toxicity. For the conditions used in this experiment, carbamoylated Hb (from urea) measured as VH may not be a sufficiently sensitive biomarker to be used for renal toxicity.

**Trial registration:**

ID 326-2008

## Background

The radiolabeled somatostatin analogue ^177^Lu-[DOTA^0^, Tyr^3^]-octreotate (^177^Lu-octreotate) is used for treatment of somatostatin receptor (SSTR) expressing neuroendocrine tumours [1-3]. Such treatments have shown promising results, e.g. longer median overall survival and higher response rate compared to chemotherapy [4,5]. However, compared to the high cure rates of ^177^Lu-octreotate therapy obtained in mice transplanted with human SSTR expressing tumours [6-8], the clinical results obtained so far are modest, and treatment of humans should be optimised.

The kidney is one of the most exposed organs after administration of ^177^Lu-octreotate, and despite the successful use of kidney uptake blocking agents such as lysine and arginine, the kidneys are regarded as the main dose-limiting organ (together with bone marrow). Based on experience from external beam radiation therapy, a tolerance dose of 23 or 28 Gy to the kidneys has been used [9]. The tolerance dose for the kidneys after radionuclide therapy is, in general, not known, but will most probably be higher than that of external irradiation due to lower dose rate, continuous irradiation and heterogeneous distributions within the organ [10]. Very few adverse effects have been presented so far. Under-treatment may then be suspected, with interruption of treatment before complete tumour remission due to feared renal toxicity.

There is a great interindividual difference in uptake and retention of ^177^Lu in the kidneys, and the mean absorbed dose per administered radioactivity may vary up to a factor of eight between patients [11]. Furthermore, it is well known that there is a high individual difference in general radiosensitivity between humans. This has been demonstrated for the kidneys after ^90^Y-[DOTA^0^, Tyr^3^]-octreotide treatment and discussed also for ^177^Lu-octreotate treatment [12]. Patients with renal risk factors, such as hypertension, diabetes, age-related decrease in renal function, but also morphological abnormalities should be treated with higher precaution [13,14].

To enable individually optimised treatment, i.e. to give each patient as much ^177^Lu-octreotate as possible without inducing nephrotoxicity, there is a need for methods/biomarkers that early indicate risk of renal injury.

^177^Lu-octreotate is mainly excreted via the kidneys. The ^177^Lu activity distribution in the kidneys is heterogeneous with the highest activity located in the cortex, especially in the proximal tubules [6,15-18]. There are several reasons for the accumulation of ^177^Lu in the tubular system: receptor-mediated endocytosis via megalin-cubilin receptors and SSTRs, uptake via amino acid/oligopeptide transporters, pinocytosis and passive diffusion. All five SSTR subtypes (SSTR1-5) are expressed both in the human and mouse kidneys [19-22].

Today the most commonly used method for estimating the impairment of the kidney function is to measure the glomerular filtration rate (GFR), which can be determined by ^99m^Tc-DTPA scintigraphy. GFR can also be estimated by, e.g. the serum creatinine level correcting for age, gender, race and body size [23,24]. The tubular extraction rate may be determined by ^99m^Tc-MAG3 scintigraphy [25,26].

Radiation may cause both short- and long-term effects on kidney function, most probably both on glomeruli and on the proximal tubules, and therefore, reduction in GFR and tubular extraction rate is not a fully adequate measure. The correlation between the radiation dose from ^177^Lu-octreotate, and the serum creatinine level seems not to be high, especially not at high absorbed doses [13,14,27]. Therefore, other biomarkers better reflecting the actual kidney damage are required.

Renal proximal tubular injury may occur before a reduction of GFR, and therefore, tubular biomarkers are needed. Retinol binding protein (RBP) is a low molecular weight plasma protein (21 kD) which is secreted by the liver and transports vitamin A in the blood. RBP is filtrated in the glomeruli and thereafter nearly completely reabsorbed in the proximal tubule cells via the megalin-cubilin receptor complex and catabolised [28,29]. Urinary RBP (RBP4 is used when analysing mice urine) might therefore be possible to be used as an early and sensitive biomarker of impairment of the reabsorption of the proximal tubular cells [30,31]. To our knowledge, no studies on urinary RBP and radiation effects on kidney function have been performed.

The serum urea level might be an alternative to serum creatinine as an indicator of reduced GFR if other impairments, for example hyper catabolism or gastrointestinal bleeding can be excluded [32]. Serum urea can be reflected by the level of carbamoylated Hb. The carbamoylated N-terminal valine in Hb can be detached from the rest of the globin chain by acidification (*in vitro*) and be measured as valine hydantoin (VH) [33-37]. VH has been proposed as a good indicator of the uremic status for patients with acute and chronic renal failure [33-35]. VH is an indicator of average urea levels over the lifetime of Hb, while serum urea gives the renal status at the moment the sample is taken. In the present work, the HPLC-MS/MS technique was introduced for quantification of VH [36,37].

The aim of this study was to investigate the potential of using urinary RBP4 and carbamoylated Hb in blood measured as VH as biomarkers of nephrotoxic effects on adult mice after ^177^Lu-octreotate treatment.

## Methods

### Radiopharmaceuticals

^177^Lu-trichloride and [DOTA^0^, Tyr^3^]-octreotate was purchased from the Nuclear Research and Consultancy Group (NRG, Petten, the Netherlands), and radiolabeling was performed according to the instructions of the manufacturer, resulting in ^177^Lu-octreotate with a specific activity of 26 MBq/μg. The fraction of peptide-bound ^177^Lu was higher than 98%, determined by instant thin layer chromatography (ITLC-SG, Pall Corporation, New York, USA) with 0.1 M sodium citrate as the mobile phase.

### Animal experiments

Six months old female BALB/c nude mice (*n* = 6/group) were injected into the tail vein with saline solution (the non-treated group), or with 60 MBq, or 120 MBq of ^177^Lu-octreotate, corresponding to a mean absorbed dose to the kidneys of approximately 21 and 42 Gy, respectively, according to the previously published data [38]. Urine was collected before injection (used as baseline values) and subsequently 14, 30, 60 and 90 days after injection. To get the volume needed for analysing RBP4 and creatinine (minimum 150 μL), spot urine samples were collected from each animal on three consecutive days at the same time each day. The last sample collection (after 90 days) was only made during one day. The urine was then frozen at −20°C until analysis.

Blood samples (0.5 to 1 ml) were taken by cardiac puncture when the animals were sacrificed (after 90 days). Erythrocytes were isolated from the whole blood, washed and thereafter frozen until further analysis of VH. One kidney from each animal was fixed in 4% paraformaldehyde for histological evaluation.

All animals had free access to food and water. The study was approved by the Ethical Committee for Animal Research at the University of Gothenburg, Sweden, Trial registration ID 326-2008.

### Analysis of urinary RBP4

RBP4 in urine samples (50 μL diluted to 100 μL) was analysed using the Mouse RBP4 ELISA kit (R&D Systems Europe Ltd., Abingdon, UK) according to the manufacturer's instructions. About 15% of the samples had to be further diluted in order to obtain a reading within the appropriate part of the calibration curve. Repeated analysis of the kit's control samples showed good precision (coefficient of variation (CV) of 3% within assay and 10% between assays).

### Analysis of urinary creatinine

To be able to correct for variations in body mass and urinary flow rate, the urinary creatinine level was determined in each urine sample. Creatinine in urine (10 μL diluted to 200 μL) was analysed using the commercially available Creatinine kit (R&D Systems Europe Ltd., Abingdon, UK), based on the Jaffe reaction, according to the manufacturer's instructions. Duplicate analysis of 12 mouse urine samples showed good reproducibility (CV 6% within assays).

### Analysis of VH in erythrocytes

**Figure 1:**
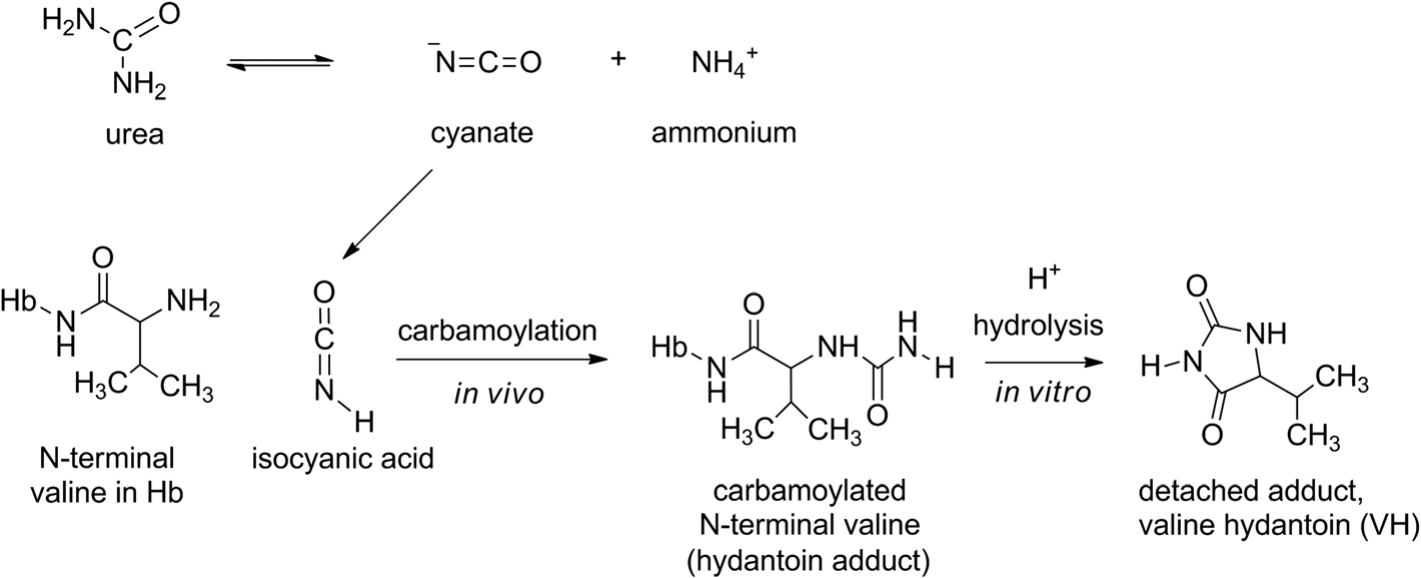
**Valine hydantoin.** Isocyanic acid is formed when urea dissociates into cyanate and ammonium. In a carbamoylation reaction, the isocyanic acid form a stable adduct with the nitrogen in amino groups, e.g. to N-terminal valine in Hb chains. The carbamoylated N-terminal can be analysed as a valine hydantoin detached by acid hydrolysis.

When urea dissociates, it spontaneously forms isocyanic acid and ammonia. In a non-enzymatic carbamoylation reaction (*in vivo*) the isocyanic acid can form stable adducts with nitrogen in amino groups, e.g. N-terminal valine in Hb chains. The adduct can be detached by acid hydrolysis and analysed as VH (5-isopropyl hydantoin) (Figure 1).

#### Sample preparation

The erythrocyte samples (100 to 400 μL) were thawed, haemolysed and mixed with a solution of acidified isopropanol. The cell residues were discarded by centrifugation, and globin was isolated through precipitation with ethyl acetate [39].

The dry globin were weighed and prepared for analysis as earlier described [40] with some modifications such as the amounts of globin and chemicals that were downscaled approximately five times. Globin from the 18 individual mice was transferred to glass tubes (4 to 16 mg/tube). A sample with myoglobin (without valine as the N-terminal amino acid) from horse skeletal muscle was added as blank reference. In addition, to measure the repeatability of the complete procedure, three samples from the same mouse (untreated) were prepared. In brief, a mixture of concentrated acids (hydrochloric acid: acetic acid) was added, and the tubes with lids were placed in 100°C for 1 h. Ammonium sulphate was added as a buffer, and the samples were neutralised with NaOH (10 M), and then the mixture was extracted twice with ethyl acetate. The organic phase was evaporated to dryness with a gentle flow of N_2_ in a heat block (60°C). The samples were dissolved in acetonitrile (AcN):H_2_O (1:1) to a concentration of 0.01 mg globin/μL, and phenylvaline hydantoin (PVH) was added as a volumetric standard to the final concentration of 0.2 μM. The samples were analysed by LC-MS/MS, and the ratio between VH and PVH was calculated. VH and PVH, used as standards, were synthesised earlier [37].

#### Instrumentation

The LC-MS/MS system consisted of a Shimadzu Prominence HPLC system coupled to an API 3200 Q trap instrument (AB SCIEX, Stockholm, Sweden). The mass spectrometer was optimised using standards of VH and PVH and operated by using an electrospray ionisation source in the positive ion mode (ESI^+^). Acquisition and data processing were done with the Analyst software version 1.5 (AB SCIEX, Stockholm, Sweden). The separation was performed with a C_18_ column (3 μm, 2.1 × 150 mm, Fortis Technologies Ltd., Neston, UK). The mobile phase system consisted of (A) H_2_O:AcN (95:5, *v*/*v*) and (B) H_2_O:AcN (5:95, *v*/*v*) both with 0.1% formic acid (FA). The gradient was 5% B for 1 min, followed by a linear increase to 25% B in 1 min, and 25% to 100% B in 7 min followed by isocratic 100% B in 3 min, with a flow rate of 120 μL/min.

The MS/MS operated in multiple reaction monitoring (MRM) mode with transitions established earlier [37] for VH m/z 143.1 > 115.0 and 143.1 > 72.1 and for the volumetric standard PVH m/z 219.2 > 120.1 and 219.2 > 72.1. The instrumental settings used for the MRM was ion source temperature 500°C, ion spray voltage 5,500 V, curtain gas (N_2_) 20, ion source gas (GS1) (N_2_) 20, collision gas (N_2_) 5 and turbo gas (GS2) (N_2_) 10 (latter four are arbitrary units from the Analyst software). Some other parameters were optimised for the different transitions and conditions used. All the samples were organised in a batch with injections of standard mixture and blank samples before, in between and after the samples. The batch was analysed for three times. Injection volume was 10 μL. The data show that the method gave repeatable results: CV was 2% to 21% for repeated analyses on three different occasions of the same sample (*n* = 18). The repeatability in the work-up procedure showed a CV of 11% of three equivalent Hb samples prepared and analysed at the same occasion. VH was not detected in the blank sample. The peaks were always >10 times the noise level.

### Morphological evaluation of the kidney tissue

The kidneys fixed in paraformaldehyde were embedded in a paraffin wax, and parallel sections (2-μm thick) were routinely stained with haematoxylin and eosin. Morphological evaluation was made by an experienced pathologist.

### Statistical analyses

Three out of 218 values of RBP4 were below the detection limit (DL) and imputed by DL/2. Five values were above the value corresponding to the highest standard used and were imputed by 1.5 times this specific value. RBP4 levels were highly skewed and log_10_-transformed before statistical analysis. As repeated samples were available for each mouse, mixed-effects models were used (PROC MIXED in SAS version 9.1; SAS Institute, Cary, NC, USA) with activity and time as fixed effects and mice as a random effect. The models also included the interaction between activity and time. The effect of activity and time after injection on RBP4 levels was analysed, and *p* values <0.05 were considered statistically significant.

The mean VH/PVH level of each group was calculated from the mean value of three repeated analyses of the same sample, for which the coefficient of variation was calculated. Student's *t* test was used to analyse data between groups, and *p* <0.05 was considered statistically significant.

## Results and discussion

### Results

#### Urinary RBP4

**Figure 2:**
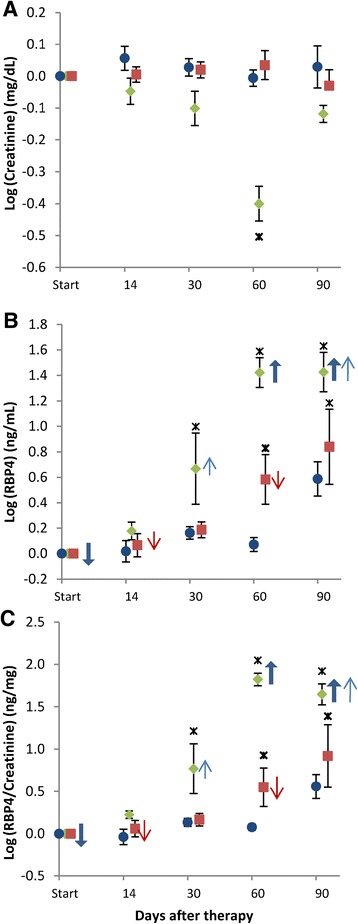
**Analysis of urinary RBP4.** Mean differences between the log_10_-transformed mean values (from three consecutive days) and the log_10_-transformed baseline mean values (from three consecutive days) for **(A)** creatinine and **(B)** RBP4 and **(C)** RBP4/creatinine, in non-treated mice (blue circle, *n* = 4 to 6), and in mice treated with 60 MBq (red square, *n* = 5 to 6) or 120 MBq ^177^Lu-octreotate (green diamond, *n* = 5 to 6) vs. time after study start. Log_10_-transformation was done since the values are skewed (see ‘Methods’). All values are shown as mean ± SEM. Upward arrows indicate that the mean RBP4 value is higher and downward arrows that it is lower than presented (see text). The heavy asterisk indicates data statistically significant different from baseline (*p* <0.05).

The urinary creatinine, RBP4 and RBP4/creatinine levels for each group are shown in Table 1. The difference between the log_10_-transformed value after treatment (14 to 90 days) and the log_10_-transformed baseline value of urinary creatinine and RBP4, and corresponding ratios (Log (RBP4/creatinine)) levels for each group, is shown in Figure 2. The reason for using log_10_-transformation is the skewness of RBP4 values. Two samples in the non-treated group at day 90, one sample at day 60 and the creatinine value at day 90 in the 60 MBq, and one sample in the 120 MBq group were not possible to evaluate correctly since the urine volume needed to perform the analyses could not be collected from these animals during three consecutive days. A few samples could not be exactly quantified regarding RBP4, because the RBP4 concentration was too high (>80 or >200 ng/mL, indicated by thin and bold upward arrows, respectively; Figure 2B) or too low (<1.25 or <6.25 ng/mL, indicated by downward thin and bold arrows, respectively; Figure 2B) to permit quantification. The samples with high RBP4 levels could not be further diluted and reanalysed since no urine remained after the first analysis.

**Table 1:** **Mean absolute urinary RBP4 and creatinine concentrations and RBP4/creatinine concentration ratios**

		**RBP4 (ng/mL)**	**Creatinine (mg/dL)**	**RBP4/creatinine (ng/mg)**
Treatment group	Days after therapy			
Non-treated	Study start	2.5 (0.2)	47 (5)	5.5 (0.7)
	14	2.6 (0.3)^b^	53 (6)	5.1 (0.7)
	30	3.5 (0.2)	50 (6)	7.5 (0.8)
	60	2.9 (0.3)^b^	46 (4)	6.6 (0.8)
	90	9.6 (2.1)	51 (5)	19 (5)
60 MBq	Study start	3.4 (0.3)	59 (6)	5.9 (0.6)
	14	4.2 (0.8)	60 (7)	7.1 (1.2)
	30	5.2 (0.5)	62 (7)	8.9 (1.2)
	60	17 (8)	68 (9)	31 (17)
	90	57 (28)	61 (11)	150 (93)
120 MBq	Study start t	3.5 (0.3)^b^	53 (4)	7 (1)
	14	5.6 (0.9)	48 (6)	12 (2)
	30	38 (20)^a^	43 (5)	94 (49)
	60	97 (17)^a^	22 (4)	480 (73)
	90	120 (31)^a^	41 (4)	350 (72)

Mean absolute urinary RBP4 and creatinine concentrations and RBP4/creatinine concentration ratios at each time-point before and after therapy for non-treated mice or mice treated with 60 or 120 MBq ^177^Lu-octreotate. Values are given as mean (SEM).

^a^RBP4 concentration too high to permit exact quantification.

^b^RBP4 concentration too low to permit exact quantification.

After 30 and 60 days in the 120 MBq group, a reduction in urinary creatinine was observed (*p* = 0.058 and *p* <0.05, respectively) compared with baseline values, while in the two other groups given ^177^Lu-octreotate, the creatinine value was relatively constant throughout the study (no significant differences between baseline and the following time points were found) (Figure 2A).

Mean RBP4 values increased with time in the 120 MBq group, and statistically significant differences were found between baseline and 30, 60 and 90 days (*p* <0.05) (Figure 2B). The RBP4 value in the 60 MBq group increased after day 30 (*p* = 0.051) and was statistically significant higher than baseline at days 60 and 90. An increased value was found in the non-treated group after 90 days.

For mean RBP4/creatinine, statistically significant differences were seen between baseline and all time points in the 120 MBq group except at day 14 *p* = 0.076 (Figure 2C). For the 60 MBq group, RBP4/creatinine was increased compared to baseline at day 30 (*p* = 0.080) and statistically significant higher at days 60 and 90. The RBP4/creatinine value increased in the non-treated group at day 90.

#### Analysis of VH in erythrocytes

**Figure 3:**
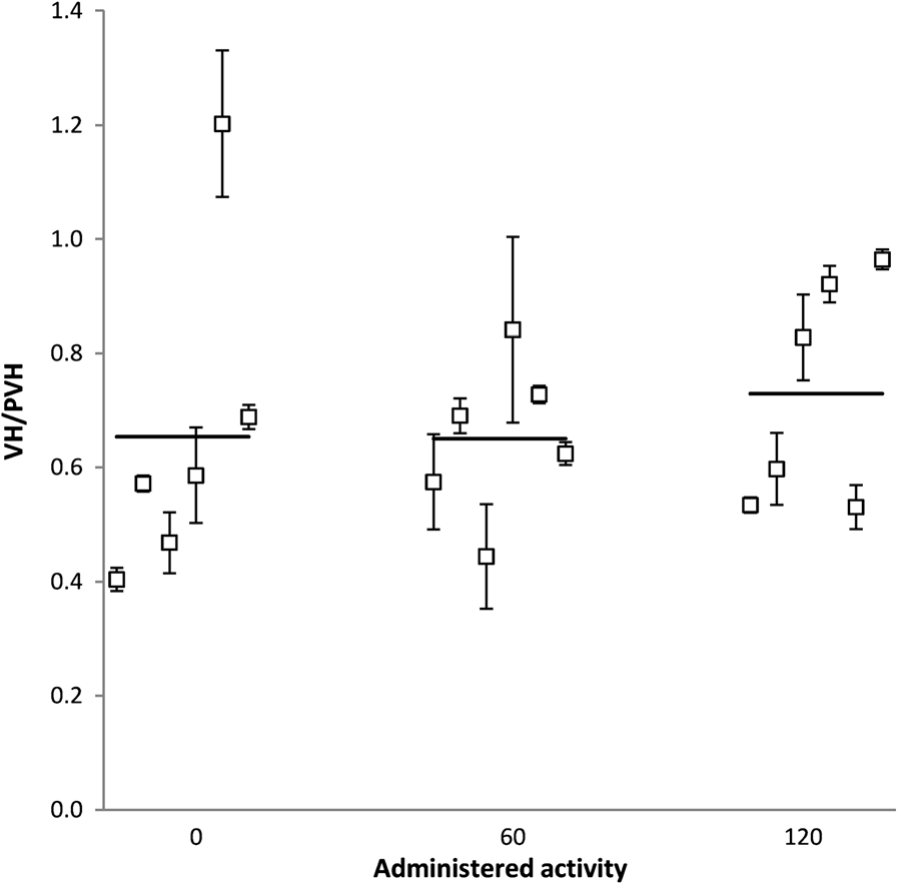
**Analysis of VH in erythrocytes.** Erythrocyte levels of VH/PVH in mice 90 days after injection of 60 MBq (*n* = 6) or 120 MBq (*n* = 6) ^177^Lu-octreotate or in non-treated mice (non-treated, *n* = 6). Each data point represents the mean value of three analyses on the same sample from one mouse. Error bars indicate SD. The horizontal lines are the mean values for each group ± SD; non-treated group: 0.65 ± 0.29, 60 MBq: 0.65 ± 0.14, and 120 MBq: 0.73 ± 0.20. Statistical analysis of the effect of treatment resulted in *p*(60 MBq) = 0.98, and *p*(120 MBq) = 0.61 compared to the non-treated group.

VH was quantified relative to the internal standard PVH (ratio between peak areas). The mean VH/PVH values (±SD) calculated for the different groups were similar for the non-treated (0.65 ± 0.29, *n* = 6) and the 60 MBq group (0.65 ± 0.14, *n* = 6), while a small increase was observed for the 120 MBq group (0.73 ± 0.20, *n* = 6) (Figure 3). There were, however, no statistically significant differences in the mean VH/PVH level between any of the groups (*p* >0.05).

#### Morphological evaluation of the kidney tissue

In all the analysed kidney tissue samples, no signs of renal injury that could be causally associated to the administration of ^177^Lu-octreotate were found. More specifically, as compared to control tissue, the glomeruli displayed no proliferative or lytic changes, the capillary loops displayed normal patency. The tubules demonstrated no signs of cellular stress or necrosis, as judged by cytoplasmal vacuolization or sloughing of plasma membrane material into the tubular lumen. The only finding that could be noted was discrete and focal signs of tubulointerstitial inflammation, where minimal nests of lymphocytes were seen grouped in the interstitium of the cortex. These changes were indeed microscopic and much dispersed. No correlation could be seen with treatment, since also the control animals displayed these changes. They are histologically regarded as a normal finding in the mouse kidney tissue. The most prominent nest of lymphocytes was demonstrated in one kidney in the control group (*n* = 1 of 6), followed by smaller areas in the 120 MBq group (*n* = 3 of 6) and in the 60 MBq group (*n* = 1 of 6).

### Discussion

In this study, we evaluated the possibility to use RBP4 and carbamoylated Hb measured as VH as biomarkers of renal toxicity after ^177^Lu-octreotate treatment on adult BALB/c nude mice. These biomarkers, to our knowledge, have not been tested in this type of application. It is important to find biomarkers that can early reflect enhanced risk of late nephrotoxicity due to irradiation, in order to optimise and individualise treatment of patients with ^177^Lu-octreotate.

In a previous study, the effects of similar amounts of ^177^Lu-octreotate were followed in the same type of mice, although at a young age (5 weeks) with longer follow-up time (6 months), but with other kidney toxicity markers [27]. In that study, serum creatinine was elevated and morphological changes in proximal tubules were obtained after administration of 90, 120 or 150 MBq ^177^Lu-octreotate, while higher urea values were obtained in the 150 MBq group after 6 months. The relative differences in weight between treated animals and control animals were similar in the present study and in the previous one, but the morphological changes were more limited in the present study, which had a lower maximum activity (120 MBq) and shorter follow-up period (3 months). Nevertheless, also an increased excretion of the low molecular weight protein RBP represents an impairment of renal (tubular) function. A similar study on rats has also been performed demonstrating similar effects [41].

In the present study, increased levels of urinary RBP4 were found after injection of 60 or 120 MBq ^177^Lu-octreotate, with higher RBP4 values and earlier elevation in the 120 MBq group. This type of dose-dependency, both regarding RBP4 level and time for increased RBP4 level, has been demonstrated earlier [27,41]. Increased urinary RBP4 levels indicate radiation-induced effects on the proximal tubules, resulting in reduced reabsorption of RBP4. In the present study, no morphological changes were seen in any of the groups, while effects on proximal tubules were seen in the previous study. This difference probably depends on the difference in follow-up time, but could also be due to the younger age (potentially higher radiation sensitivity) and smaller kidneys (resulting in higher absorbed dose) in the previous study. Thus, RBP4 may be used as an early biomarker for radiation-induced late nephrotoxicity.

In general, we did not find consistent changes in urinary creatinine levels, but the decrease in urinary creatinine at 30 and 60 days in the 120 MBq group may be caused by a decreased GFR. If this is the case, creatinine adjustment of RPB values is inappropriate. When taking spot urine samples, the urine creatinine levels are used to adjust for differences in urinary flow rate, making urine more or less concentrated, since urinary creatinine excretion is relatively constant during the day [42,43]. Urinary creatinine reflects the concentration of fluid passing through the glomerulus and is only to a small extent affected by tubular reabsorption or secretion. In this study, RBP4/creatinine values did not add any new information compared to RBP4 values alone.

For VH used as marker for urea, no statistically significant increase was observed in any of the groups 90 days after ^177^Lu-octreotate administration. Only a small, non-significant increase was indicated for the 120 MBq group compared with the non-treated group. One explanation for the lack of significant changes in VH level in this case was probably that the renal injury was not severe enough to cause sufficient changes compared to the background level of VH; the steady-state background level would correspond to 20 days accumulation of carbamoylated Hb in mouse; cf. [44]. A lower CV for the analytical method for VH, e.g. through the use of isotope-substituted VH as internal standard, would further improve the possibilities to detect changes in urea concentrations. The introduction of LC-MS/MS for analysis of VH is a step forward compared to the earlier used less specific HPLC analysis. Another explanation may be low effects on urea level due to irradiation, which might be supported by our previous findings [27,41]. It should be noted that the lifetime of erythrocytes and these adducts in mouse is approximately 40 days, why it is not unrealistic to obtain higher levels already after 90 days.

In the present study, the mean absorbed dose to the kidneys was approximately 21 and 42 Gy after injection of 60 and 120 MBq ^177^Lu-octreotate, respectively, assuming homogeneous activity distribution [38]. It is known that the ^177^Lu distribution is heterogeneous with the highest uptake in the kidney cortex [45]. Due to the relatively small size of the mouse kidney, the range of the electrons emitted by ^177^Lu will result in a relatively homogeneous exposure of the entire kidney. In our previous study on nude mice, the absorbed dose to kidney cortex was 47 Gy after administration of 120 MBq ^177^Lu-octreotate [27], which is only about 10% higher than the mean absorbed dose to the kidney from homogeneous ^177^Lu distribution.

The results indicate a possibility to use RBP as an early responding marker of late renal impairment after ^177^Lu-octreotate treatment in humans. It should be noted that urinary RBP4 level only indicates impairment of proximal tubular cells and may not reflect effects on the other parts of the kidney. Before RBP can be used in clinical routine, studies correlating urinary RBP excretion kinetics with absorbed dose to the kidneys and risk of renal toxicity must be performed. The lowest RBP detection level should be determined and correlated to absorbed dose to the kidney (cortex). Furthermore, it is also important to establish the relationship between absorbed dose to the kidneys from ^177^Lu-octreotate administration and the severity of renal toxicity on a short- and long-term basis. There are several low molecular weight proteins, such as beta-2-microglobulin (B2M) and alpha-1-microglobulin (A1M) that also could serve as urinary biomarkers of renal tubular damage in humans, but RBP has the advantage of better stability in urine than B2M and longer experience of normal levels in urine than for A1M.

Further long-term studies on mice are ongoing not only to validate if RBP4 may be efficient in predicting late renal toxicity at lower absorbed doses and for fractionated administration but also to investigate other biomarkers of proximal tubular and glomerular damage in serum and urine. These studies have larger group size and a longer follow-up period (up to 1 year). Clinical studies are also planned.

## Conclusions

Urinary RBP4 is a promising new biomarker for radiation-induced renal toxicity. Higher absorbed dose to the kidneys resulted in higher RBP4 excretion occurring earlier in time. Measurement of VH, marker for urea in blood, was either not sensitive enough to detect differences in urea concentrations due to renal toxicity during the conditions applied in this work or urea is not suitable for early detection of renal impairment.

## Abbreviations

^177^Lu-octreotate: ^177^Lu-[DOTA^0^, Tyr^3^]-octreotate
CV: Coefficient of variation
DL: Detection limit
GFR: Glomerular filtration rate
Hb: Haemoglobin
MRM: Multiple reaction monitoring
PVH: Phenylvaline hydantoin
RBP: Retinol binding protein
SSTR: Somatostatin receptor
VH: Valine hydantoin
